# Predictors of infant-survival practices among mothers attending paediatric clinics in Ijebu-Ode, Ogun State, Nigeria

**DOI:** 10.1186/s12889-020-09310-3

**Published:** 2020-08-17

**Authors:** Eniolufolake Elizabeth Sokefun, Nnodimele Onuigbo Atulomah

**Affiliations:** 1 College of Public Health, Kent State University, Kent, OH USA; 2 Department of Health Science, Cavendish University, Kampala, Uganda

**Keywords:** Health-literacy, Social-support, Self-efficacy, Infant-survival practices, Nursing mothers

## Abstract

**Background:**

Despite concerted global efforts towards achieving infant-survival, infant mortality lingers as a problem in developing countries. Environmental and personal-level factors are assumed to account for this situation. This study was undertaken to provide better understanding of the dynamics of predictors of infant-survival practices among mothers with infants attending paediatric clinics.

**Methods:**

A cross-sectional survey design was adopted. Data was collected from 386 nursing mothers selected by convenience sampling. Interviewer-administered questionnaires were used for data collection. The questionnaire consisted of 38-items including demographic information of respondents, health-literacy counsels received during antenatal care, social-support from significant others, and self-efficacy to carry-out infant-survival instructions. Responses were transformed into rating scales for each variable and data analysis was conducted by linear regression analysis with test of hypotheses at 5% level of significance.

**Results:**

The mean age of respondents was 29.8 ± 5.8 years. Majority (81.6%) were married. Yorubas (83.90%) were predominant. Participants had mean scores of 10.50 ± 3.83, 10.56 ± 3.70 and 16.61 ± 4.56 respectively computed for levels of health-literacy, social-support, and self-efficacy. The dependent variable measured level of infant-survival practices and respondents scored 16.53 ± 4.71. The study found a significant association among variables. Self-efficacy was the major predictor variable of self-reported infant-survival practices (R = 0.466; R^2^ = 0.217; *P*<0.05).

**Conclusion:**

We conclude that participants had average levels of health-literacy, social-support, self-efficacy, and infant-survival practices. Healthcare providers should make efforts to empower pregnant women on activities essential for infant-survival. Family members of nursing mothers should as well be knowledgeable about the advantages of supporting them.

## Background

Global death rates of children under 5 years of age from 1990 to 2015 showed marginal improvement towards achieving the target for the Millennium Development Goal-4 with records of 90.6 to 42.5 deaths per 1000 live births [1]. However, from reports, the global percentage reduction in infant and child mortality was 53% compared with the 15-year goal which was aimed at 75% reduction [2]. Similarly, data revealed the mortality rate of children under 5 years in West Africa to be 98.7 deaths per 1000 births, which is about fifteen times the average values for developed regions [1]. Specifically, statistics from 2015 revealed that 69 out of 1000 infants in Nigeria died [3]. Among the 5.941 million children who died in 2015 before their fifth birthdays were 2.681 million neonates [4]. These figures are pointers to the non-attainment of the MDG-4.

Some causes of infant and child mortality are preterm birth complications, infections, pneumonia, malaria [3, 5], diarrheal diseases [6], tetanus, measles, meningitis, birth asphyxia, poor feeding, HIV/AIDS and injuries [7]. For example, acute respiratory infections such as pneumonia account for millions of infant and child deaths annually from sub-optimum feeding and lack of immunisation [8]. Emphatically, research indicates that certain overlooked environmental-level factors that contribute to infant mortality are inadequate antenatal attention, poor service provision from healthcare workers, and absence of skilled-care providers [9]. Requirements of mothers include skill-building on health education and the relevance of timely immunisation [10], exclusive breastfeeding [11], and prevention of diarrhoea and malaria. If not critically attended to, these issues will continually contribute to infant deaths regardless of the Sustainable Development Goals recently initiated.

Fehling et al (2013) asserted that a link exists between the skilfulness of health care providers and maternal and child health outcomes [12]. Behaviour-change in mothers to enhance their self-efficacy in ascertaining infant-survival will be because of comprehensible counselling [13], clear health education messages and skilled delivery [14, 15]. Adebowale and colleagues (2012) observed that socio-demographic attributes such as age and educational status of mothers affect infant care, with children born in maternal and child health-facility deprived areas being more likely to die than those born in better established places [16]. Although, infant mortality is more evident among poorer and less educated mothers [16], behaviours can influence infant health outcomes among highly educated mothers. Similarly, access of mothers to well-equipped health facilities with skilled health providers, proper sanitation and clean water impact infant-survival. Thus, it is relevant to understand the dynamics of infant-survival within these personal and environmental contexts.

Importantly, deficiencies emerging from lack of social-support exacerbate poor health outcomes. Programs with innovative approaches to engage key influencers (such as fathers and grandmothers) to assist and encourage mothers would be more successful in influencing their behaviours to improve infant care, including infant feeding [17]. Research has identified health-literacy, social-support, and self-efficacy as personal and environmental-level components for the enhancement of infant-survival practices. For example, Fry-Bowers and colleagues (2014) inferred that health-literacy has a positive influence on decision-making for mothers regarding infant care [18]. Moon et al (2016) concluded that the multi-level approach for infant care should include regulation of policies, modification of cultural and ethnic values, education skills and health counsels by professionals [19].

Infants are a delicate and vulnerable sub-population; their survival is a fundamental pointer to maternal and child health and the development of any nation [20, 21]. Therefore, we sought to identify to what extent would demographic characteristics, health-literacy, social-support, and self-efficacy predict infant-survival practices among an almost average educated sample of mothers with infants attending paediatric clinics. We hypothesized that the predictors will be significantly associated, and one of the predictors will exhibit greater influence on infant-survival practices.

## Methods

### Study design and participants

This study adopted the cross-sectional survey design. It was conducted in Ijebu-Ode local government situated in Ogun state, South-West Nigeria. The local government has twelve primary health centres and one tertiary health facility. We randomly selected nine primary healthcare centres that cut across all the wards in the local government. We then included the only tertiary health facility in the study location. The population was 2006 mothers whose infants were receiving postnatal care at the time of the study. We estimated the sample size to be 423 using Cochran’s formula for sample size computation [22]. We anticipated an attrition probability of 10%. Overall, from the ten health facilities, 386 consenting nursing mothers drawn by convenience sampling were enrolled into the study. Because the number of postnatal attendees for each health facility depends on the level of patronage and the population in the locality, none of the facilities recorded similar number of respondents.

### Instrument and data collection

We adopted the educational and ecological assessment phase (Predisposing, Reinforcing, and Enabling factors) of the PRECEDE model [23], bearing in mind that these parts of the framework are causally linked to expected behaviour. We first developed the instrument in English and then translated it to Yoruba language because Ijebu-Ode is home to mostly people of the Yoruba ethnicity (a major Nigerian ethnic group inhabiting states in the Southwestern part of Nigeria). Thus, respondents could select the questionnaire written in the language option suitable for them. We initially conducted a pilot-test for internal consistency of the instrument using 40 nursing mothers from Ilishan primary health centre (25 km from study site), followed by a re-test for reliability of the instrument with the same participants. Data from the pilot-test was statistically analysed and a Cronbach alpha standard score of 0.738 was obtained with corrections made where necessary. We collected data from participants by interviewer-administered technique from the 27th of February 2017 to the 21st of March 2017.

### Constructs from the PRECEDE framework

**Fig. 1:**
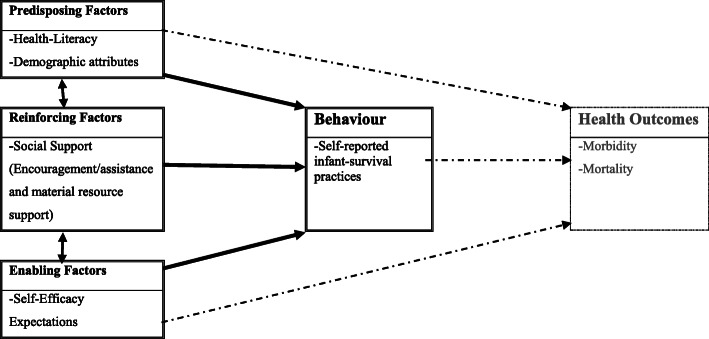
Variables in the educational and ecological assessment phase of the PRECEDE meta-model expressed in the study objectives demonstrating linkages between predisposing, reinforcing, enabling factors and infant-survival practices

*Predisposing factors* are personal-level attributes that motivate behaviour prior to or during the occurrence of that behaviour. For this study, the predisposing factors we assessed were health-literacy and demographic characteristics of respondents. *Reinforcing factors* are environmental-level influences that stem from repetitive emphasis laid on the behaviour of interest. For this study, we assessed social-support from family members as the reinforcing factor. *Enabling factors* are the characteristics of the environment that facilitate action and any skill or resource required to attain the specific behaviour. These may be programs, services, availability and accessibility of resources, or new personal skills. The enabling factor for this study was self-efficacy. We described the *behaviour* of concern as infant-survival practices. Figure 1 describes the linkages between the variables as adopted from the PRECEDE Framework.

### Variables

*Demographic data* elicited information on age, marital status, occupational status, religion, ethnicity, educational attainment, parity, and number of infants ever lost. As Renkert and Nutbeam [24] previously described, we defined maternal *health-literacy* as cognitive and social skills that determine mother’s motivation and ability to gain access to, understand, and apply information in ways that promote and maintain their health and that of their children. The variable contained items such as instructions about sterilization of infants’ items, infant feeding, exclusive breastfeeding and use of Insecticide Treated Nets. Two items had the dichotomous response format (Yes/No) and were scored at a point each. Six items had multiple-choice response format and were scored at 16 points; each right response being scored at one point. Thus, health-literacy was measured on 18-points rating scale. The *social-support* variable included questions on the frequency of receiving assistance and encouragement to practice health counsels and assistance with infant health and care from family members. It consisted of five items with the Likert-type 4-response options (*never, rarely, occasionally, and always; the least being 0 and the highest being 3*) and 1-item with multiple-choice response pattern rated at two points. Thus, the social-support variable was measured on a 17-point rating scale. We measured s*elf-efficacy* as self-responsibility, confidence and willingness of mothers to adhere to infant-survival instructions. The variable consisted of eight items with the Likert-type 4-response option (*strongly agree, agree, disagree, and strongly disagree; the least being 0 and highest being 3*). Hence, we rated self-efficacy on a 24-point rating scale. The dependent variable was i*nfant-survival practices* which assessed mothers’ behavioural adherence to antenatal health instructions, prevention of malaria, practice of exclusive breastfeeding, and health appointment keeping. The variable included seven items with Likert-type 4-response options (*never, rarely, occasionally and always; the least being 0 and the highest being 3*) and was measured on a 21-point rating scale.

### Analyses

The ratings of the variables provided responses to the research questions on the levels of health-literacy, social-support, self-efficacy and infant-survival practices of respondents. Data derived were computed and analysed using the Statistical Package for Social Sciences (SPSS) version 21. Responses from the variables were transformed into rating scales to derive standard measures. Correlation and linear regression analysis were conducted to give statistical responses to the research hypotheses. Analysis of Variance was evaluated to assess how demographic data influenced responses of participants on infant-survival. Decision rules for the test of null hypotheses were set at 5% level of significance. Therefore, *P*-Values greater than 5% were rejected.

## Results

### Sample characteristics

From the ten health facilities, 386 nursing mothers participated in the study. The mean age of respondents was 29.8 years (SD = 5.8; 95% CI = 29.2–30.4) with the youngest being 16 years while the oldest was 43 years. The modal age was 30 years (7.8%; *n =* 30). Majority were Yorubas (82.9%) while other ethnicities (Idoma, Ishan, Okene, Delta, Ijaw, Edo, Eghele and Coutonou) consisted 3.4% of the sample. Further, 81.9% were married, 58.0% were self-employed and 65% were Christians. In addition, 42.5% had attained tertiary education and 33.4% had more than two children. Forty-six (11.90%) had lost one or more infant(s) before the survey. Itamapako, the most rural ward scored the least mean value on infant-survival practices (12.7). Upon assessing mean scores for infant-survival practices, the following reported better practices: younger respondents (17.2), the single and married (16.6), civil servants/private sector workers (16.7), Yorubas/Hausas (16.7), respondents who had attained tertiary education (17.8), those who had two children (16.6), and respondents who had not lost any child (16.8). Table 1 describes the overall demographic characteristics of respondents and the mean values for reporting infant-survival practices with 95% confidence intervals.

**Table 1:** Demographic characteristics of participants

Items	Number of Respondents = 386		Mean scores and 95% CI on Infant-survival practices
	Frequency	Percentage	
Health Centres			
Ikanigbo/Isoku	35	9.1	15.6 (13.7–17.5)
Italapo	49	12.7	16.5 (15.1–17.9)
Itamapako	21	5.4	12.7 (11.0–14.4)
Itantebo	19	4.9	18.6 (17.6–19.6)
Ita-Osu	38	9.8	16.7 (15.0–18.4)
Iwade Oke/Isale	45	11.7	17.2 (16.1–18.3)
Odo-Esa	77	20.0	16.0 (14.7–17.3)
Oke Oyinbo	52	13.5	16.6 (15.3–17.9)
Oke-Aje	24	6.2	19.2 (18.1–20.3)
Otunba Tunwase National Paediatric Centre (Tertiary)	26	6.7	16.9 (15.7–18.1)
Age			
16–25	97	25.1	17.2 (16.3–18.0)
26–35	221	57.3	16.2 (15.5–16.8)
36 and above	68	17.6	16.9 (15.8–18.0)
Marital status			
Single	60	15.6	16.6 (15.4–17.7)
Married	315	81.6	16.6 (16.1–17.1)
Separated	7	1.8	13.0 (8.1–17.9)
Widowed	2	0.5	16.0 (−47.5–79.5)
Divorced	2	0.5	16.0 (−22.0–54.0)
Occupational Status			
Housewife	32	8.3	16.5 (14.6–18.4)
Unemployed	57	14.8	16.3 (15.1–17.5)
Self-employed	224	58.0	16.5 (15.8–17.1)
Civil servant/Private firm	73	18.9	17.0 (16.1–17.9)
Religion			
Christianity	252	65.3	16.7 (16.1–17.3)
Islam	122	31.6	16.6 (15.7–17.5)
Traditional belief	12	3.1	12.5 (9.0–16.0)
Ethnicity			
Yoruba	320	82.9	16.7 (16.2–17.2)
Igbo	41	10.6	16.1 (14.3–17.9)
Hausa	12	3.1	16.7 (14.2–19.2)
Other ethnic groups	13	3.4	14.2 (11.4–17.0)
Educational attainment			
Non-formal	21	5.4	12.8 (9.5–16.1)
Primary	37	9.6	14.8 (13.4–16.2)
Secondary	164	42.5	16.2 (15.4–16.9)
Tertiary	164	42.5	17.8 (17.2–18.4)
Number of Children alive			
One child	124	32.1	16.4 (15.5–17.3)
Two children	133	34.5	16.6 (15.8–17.4)
More than two children	129	33.4	16.5 (15.7–17.3)
Number of infants ever lost			
None	340	88.1	16.8 (16.3–17.3)
One	31	8.0	14.0 (12.1–15.9)
Two	13	3.4	15.7 (11.4–20.0)
More than two	2	0.5	12.5 (6.1–18.9)

### Health-literacy

The study observed that an above average of the respondents (55.2%) understood that infants are fragile and cannot be fed with just any food. Majority (89.6%) reported that handling the food of infants in unclean ways will lead to diarrhoeal infections. Only 15.5% affirmed that dirty environments, mosquito bites, and herbal concoctions are very harmful to infants while 10.9% added that colostrum and immunisation are harmful to the infant. Less than a quarter (18.7%) admitted that breastmilk is the best diet for an infant that is less than 6 months, others (81.3%) included infant formula and pasteurized milk. When asked for the activity that is relevant for the wellness of infants, 21.8% chose antenatal and postnatal care sessions.

Also, 40.7% of respondents affirmed that keeping the environment clean and cleaning the nipple before breastfeeding will protect their infants from falling ill. Not more than 27.7% claimed to have received counsels on all three options which included the use of insecticide-treated nets (ITNs), exclusive breastfeeding (EBF) and alcohol avoidance. The analysis of the data showed that about a quarter (22.3%) of the participants reported ability to carry-out all the five infant care activities (administration of prescribed infant medications, use of ITNs, preparation of infant meals, preparation of oral rehydration solution and sterilization of infants’ items) without aid. Table 2 summarizes the responses of participants on health-literacy.

**Table 2:** Health-Literacy responses of respondents

Items	Number of Respondents in this study = 386	
	Frequency (N)	Percentage (%)
Infants are fragile and cannot eat just any food.		
Yes	213	55.2
No	173	44.8
When the food given to an infant is handled in an unclean way, it can lead to diarrhoeal infections		
Yes	346	89.6
No	40	10.4
Which of the following should be prevented because it is very harmful to an infant (*mosquito bites*, *dirty environments, herbal concoction*, *colostrum, immunisation*)		
Mosquito bites, dirty environments, herbal concoction	60	15.5
Two correct responses	135	35.0
One correct response	149	38.6
All options or wrong responses	42	10.9
Which of these is the best food for infants who are less than 6 months old? (*infant formula, breast milk, pasteurised cow milk*)		
Breastmilk only	72	18.7
All options or wrong responses	314	81.3
For the wellness of an infant, choose the activity that is relevant from the list below. (*antenatal sessions, postnatal sessions, creating dimples*)		
Antenatal and postnatal sessions	84	21.8
Antenatal or postnatal sessions	245	63.4
All responses	57	14.8
Which of these is an essential sanitary practice to ensure that the infant is protected from falling ill? (*keeping the environment clean, bathing the infant with adult medicated soaps, cleaning the nipple before breastfeeding*)		
Keeping the environment clean and cleaning the nipples before breastfeeding	157	40.7
Keeping the environment clean or cleaning the nipples before breastfeeding	168	43.5
All responses	61	15.8
Which of the following did you receive instructions/counsels about during antenatal care? (*use of insecticide treated nets, exclusive breastfeeding, avoidance of alcohol consumption*)		
All three responses	107	27.7
Two responses	97	25.1
One response	169	43.8
No response	13	3.4
Which of the following activities can you carry out without assistance? (*administration of prescribed medication for my infant, use of insecticide-treated net, preparation of infant food, preparation of oral rehydration solution, sterilisation of my infant’s items*)		
All five responses	86	22.3
Four responses	47	12.2
Three responses	50	13.0
Two responses	48	12.4
One response	139	36.0
No response	16	4.1

### Social-support

Ninety-four participants (24.4%) reported having no one to care for them and their infant. About average (48.4%) affirmed that they always got assistance from their husbands when taking their infants for immunisation. Minority (5.4%) reported that when taking their baby for immunisation, their family members tell them immunisation is not necessary for their baby. Less than a quarter (24.1%) always had someone to assist them in taking their infants for clinic sessions. Less than average (44.0%) always received encouragement to practice health counsels by those around them while 37.8% always had someone to take care of them and their infant. Table 3 gives details of respondents’ responses on social-support.

**Table 3:** Social-Support responses of participants

Items	Frequency/Percentage			
	*Never*	*Rarely*	*Occasionally*	*Always*
I get assistance from my husband when taking my infant for immunisation	75 (19.4)	54 (14.0)	70 (18.1)	187 (48.5)
When it is time to go for immunisation, my family members tell me it is not necessary for my baby.	309 (80.1)	39 (10.1)	17 (4.4)	21 (5.4)
How often do you have someone to assist you when taking your infant for other clinic sessions?	96 (24.9)	100 (25.9)	97 (25.1)	93 (24.1)
I am encouraged to practice the health counsels I have received for my infant by those around me.	78 (20.2)	68 (17.6)	70 (18.1)	170 (44.1)
How often do you have someone else to take care of you, and your infant?	86 (22.3)	68 (17.6)	86 (22.3)	146 (37.8)
**Number of Respondents = 386**				
	**Frequency**	**Percentage**		
Who is usually present with you in ensuring the welfare of your infant?				
More than one person	39	10.1		
Either of my husband/mother/mother-in law/my sibling/husband’s sibling	253	65.5		
No one	94	24.4		
**Number of Respondents = 386**				

### Self-efficacy

Below a fifth of the participants (14.8%) were not confident taking infants for clinic sessions, less than average (39.1%) were willing to comply with 6 months EBF instructions, 14.0% were not confident taking their infants for immunisation, and 50.8% strongly agreed to use ITNs to prevent their infants from being infected with malaria. Likewise, 45.3% strongly agreed that if they had their way, they will wash their hands frequently when they handle their infant’s items, 33.7% strongly disagreed that sterilizing infant’s objects before using them is inconvenient, and 13.0% strongly agreed to not attending antenatal sessions in the future. Few of the participants (11.7%) reported finding it tasking to clean their environment all the time. Table 4 describes respondent’s self-efficacy.

**Table 4:** Self-efficacy of respondents to adhere to infant-survival instructions received

Items	Number of respondents = 386 Frequency/Percentage			
	*Strongly Agree*	*Agree*	*Disagree*	*Strongly disagree*
I am not confident to take my infant for clinic sessions	57 (14.8)	61 (15.8)	102 (26.4)	166 (43.0)
I am willing to comply with the counsel on 6 months exclusive breastfeeding for my baby	151 (39.1)	156 (40.4)	41 (10.6)	38 (9.9)
I am not confident enough to take my infant for immunisation	54 (14.0)	43 (11.1)	109 (28.3)	180 (46.6)
From what I know about malaria, I will be careful to use the insecticide treated net to protect my infant from getting it	196 (50.8)	137 (35.5)	17 (4.4)	36 (9.3)
If I have my way, I will wash my hands frequently when I need to touch my infant’s items	175 (45.3)	165 (42.8)	24 (6.2)	22 (5.7)
Sterilizing objects before using them for my infant is inconvenient	56 (14.5)	103 (26.7)	97 (25.1)	130 (33.7)
Antenatal sessions are time-consuming, I will not attend them in the future	50 (13.0)	70 (18.1)	105 (27.2)	161 (41.7)
Keeping my environment clean all the time is tasking to do.	45 (11.7)	54 (14.0)	83 (21.5)	204 (52.8)
**Number of respondents = 386**				

### Infant-survival practices

Majority (65.3%) reported to always practice 6 months EBF, 63.0% reported that they always made use of ITNs, 73.8% reported always taking their infants for immunisation when due, and 47.7% always cleaned their nipples before breastfeeding their infants. Above average (58.0%) claimed to sterilise their infant’s items before use, 61.4% reported that they always took their infants for clinic check-ups when required, and 83.9% reported always keeping their environment clean. Table 5 describes the responses of participants’ infant-survival practices.

**Table 5:** Infant-survival practices

Items	Frequency/Percentage			
	*Never*	*Rarely*	*Occasionally*	*Always*
I practice 6 months exclusive breastfeeding for my baby	45 (11.7)	53 (13.7)	36 (9.3)	252 (65.3)
I make use of Insecticide Treated Nets to prevent my baby from getting malaria.	30 (7.8)	61 (15.8)	52 (13.5)	243 (62.9)
I take my baby for immunisation when due.	23 (6.0)	30 (7.8)	48 (12.4)	285 (73.8)
How often do you clean your nipples when you need to breastfeed?	44 (11.4)	75 (19.4)	83 (21.5)	184 (47.7)
How frequently do you sterilize the items used for your infant?	36 (9.3)	65 (16.9)	61 (15.8)	224 (58.0)
I take my infant for regular clinic check-ups when required.	25 (6.5)	44 (11.4)	80 (20.7)	237 (61.4)
I keep my environment clean to protect my baby from falling ill.	11 (2.9)	20 (5.2)	31 (8.0)	324 (83.9)
**Number of Respondents = 386**				

### Variable scores

On an aggregate weighted 18-point reference scale, respondents scored a mean of 10.50 in health-literacy. On a scale of 17-points, respondents scored an overall mean of 10.56 in social-support. The aggregate weighted score for self-efficacy of respondents was 16.61 on 24-point rating scale. In addition, the mean scores for respondents’ infant-survival practices was 16.53 on 21 points rating scale. Table 6 gives a summary of respondent’s scores for each variable.

**Table 6:** Mean scores of Respondents

Variables	Number of respondents = 386			95% Confidence Interval
	Score on rating scale	Mean (SE) ± SD	Percentage (%)	
Health-literacy	18	10.50 (0.19) ± 3.83	58.33	10.12–10.88
Social-support	17	10.56 (0.18) ± 3.70	62.11	10.19–10.94
Self-efficacy	24	16.61 (0.23) ±4.56	69.20	16.16–17.07
Self-reported infant-survival practices	21	16.53 (0.24) ±4.71	78.71	16.06–17.01

### Relationship among variables

**Fig. 2:**
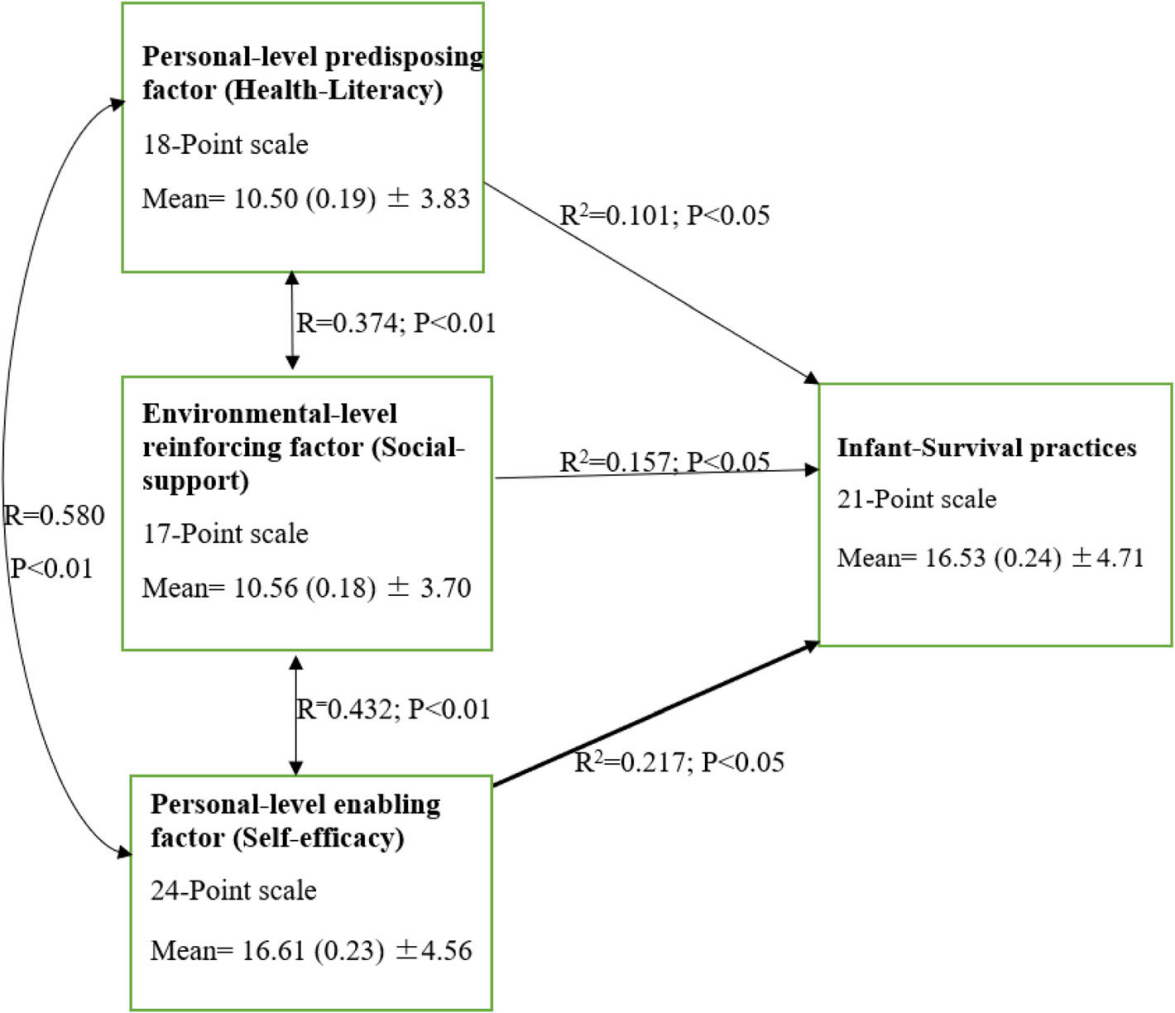
Figure showing relationship between variables

In accordance with the hypotheses postulated in this study, a regression analysis showed that there was a relationship between the independent and dependent variables. Further analysis revealed that self-efficacy was the major predictor of infant-survival practices (R = 0.466; R^2^ = 0.217; *P* <0.05). Pearson’s correlation revealed that there was a positive correlation between variables in the conceptual framework. Bivariate analysis was significant at the 0.01 level (2- tailed). Figure 2 reports the regression coefficients and corresponding *p*-values of statistical relationship.

## Discussion

Studies to investigate infant-survival should be geared towards understanding why infant mortality lingers in developing nations and how it can be significantly addressed. This study assessed ways by which demographic attributes, health-literacy, social-support and self-efficacy of nursing mothers explain infant-survival practices. The findings lucidly indicate that these factors are imperative in ensuring the survival of infants. Results obtained showed greater mean scores between infant-survival practices and better marital relationships, lucrative occupations, religion, and tertiary learning. A considerable proportion (11.90%) of the respondents had lost an infant or more before the survey was conducted.

Some participants denied that giving an infant herbal concoction will be harmful. Many disagreed that breastmilk is the best food for an infant less than 6 months. A good number of respondents did not take cleaning of the nipple before breastfeeding as an essential sanitary practice and could not carry-out all the instructions listed for infant-survival. Some reasons for these negative responses could be poor antenatal sessions or lack of support in carrying-out these practices when assistance was needed. Contrary to findings by Bolam and colleagues (1998) [25] which opined that health information and counselling had no positive impact on infant care practices, this study revealed a significant association between health-literacy of mothers and infant-survival practices (R^2^ = 0.101; *P*<0.05). This result is however consistent with some studies that suggested that behaviour-change geared towards infant care and reduction of infant mortality can be achieved by health education and counselling of caregivers of infants [14, 15]. These recent findings may be because of changes that have occurred in these domains over time.

Assistance from family members play a pivotal role in the ability to decipher and act on health information received. Among participants, less than average (48.4%) consistently received encouragement and assistance from their husbands to take their infants for immunisation. Only a few participants always got assistance for self and infant care from family members while some were dissuaded to immunise their infants. The positive relationship between social-support and infant-survival practices (R^2^ = 0.157; *P*<0.05) has been traced to relevant literature. A study [26] stated that involvement of men during pregnancy and childbirth is significant in the safety of the mother and child through emotional, physical and financial support, hence, men should equally receive health education for infant care. Similarly, Mukuria et al (2016) [17] resolved that key influencers such as fathers and grandmothers should be engaged in support for recommended infant care practices.

Reports on self-efficacy showed that an above average of the participants were willing to comply with the 6 months EBF practice. However, some reported finding it tasking to clean the environment regularly while some were not confident to take infants for immunisation, sterilise infant’s items or attend antenatal sessions during a future pregnancy because they are usually time consuming. The relationship between self-efficacy and infant-survival practices (R^2^ = 0.217; *P*<0.001) can be linked to access to and comprehension of health-literacy counsels, availability of assistance in carrying-out health instructions, and determinism. For example, a nursing mother may find it tasking to always clean the environment if she has no assistance.

Further analysis showed that Itamapako, the most rural setting scored poorest on infant-survival. Rural areas have less facilities, poor quality of healthcare, and are underprivileged. As portrayed in this study, similar studies have shown that higher infant mortality rates are predominant in rural areas due to poor facilities, low socioeconomic status, and scarce attention from the few health attendants available [27, 28]. Furthermore, married, and single mothers scored high in infant-survival. The presence of a marital partner aids collective care. Husbands of such women will not only assist in catering for the infants but also encourage their wives to go for antenatal sessions, offer financial and tangible support and make them intentional to self-efficacy. The divorced, separated and widowed may not have such assistance. Single mothers may be accustomed to living alone and being able to cater for themselves without someone else being around.

Participants engaged in lucrative jobs reported better outcomes because they are more likely to be educated, empowered, and funded than the unemployed, housewives and self-employed. The self-employed in this study referred to those who engaged in small scale businesses or petty trading. On examining the effects of employment on infant mortality, Ko and colleagues (2014) [29] showed related results to this and asserted that employment ensured tangible support for the mother and improved her self-efficacy to carry-out infant care counsels. A possible justification for the high scores of Christians and Muslims and low outcomes of those of the traditional religion could be that the traditional believers rely less on clinic instructions but more on herbs and may not have been consistently attending antenatal sessions. From results on ethnicities of participants, other ethnic groups may have reported lower scores because they did not have their family members around and are the least likely to be educated or employed with a lucrative job.

This study also found a higher infant-survival score among participants who had attained tertiary education as compared to those of lower educational status. Infant mortality is associated with poorer regions where women are hardly educated [30]. People who attain higher levels of academic learning are more likely to understand the depth of health-literacy instructions and may be able to tell their family the exact assistance needed for better support and infant care. Adebowale, Yusuf and Fagbamigbe (2012) [16] similarly found in a study that lower mortalities were observed among individuals who were more educated and engaged more in profitable jobs.

Although, we found no difference in means scores of respondents regarding parity, mothers with history of previous infant deaths scored poorer in infant-survival practices. Lack of essential elements for infant care may have accounted for the previous infant mortalities. While 42.5% of participants had attained some form of tertiary education, other covariates may be responsible for low infant-survival practices. For example, traditional belief and employment status may negate positive outcomes. Similarly, mothers who have attained tertiary learning and who are also employed with better jobs may be hindered from dedicating adequate time to infant care practices such as EBF. This implies that infant-survival should be tackled from the multi-level perspective as it is not a function of one factor.

This study is not without limitations. First, the nursing mothers considered for the survey were those whose infants were attending health centres for immunisation. We were unable to do a closer community-based study, hence, those who were not attending immunisation sessions were not considered. The results may therefore not be generalized for all nursing mothers. Secondly, results based on ethnicity may be favourable to the Yorubas because they comprised the largest ethnic proportion of the location. Thirdly, respondents may have been bias in giving responses since the data retrieved were based on self-reported information. Despite these limitations, this study addresses key issues in a suburban setting and relates the findings to all personnel involved in the prevention of infant morbidity and mortality.

## Conclusion

The ability of mothers to carry-out infant-survival practices does not depend on a single activity. From this study, it has been established that the multi-level approach from personal to environmental-level factors of nursing mothers will collectively have positive behavioural effects on infant-survival practices based on the theories adopted to conceptualize the problem phenomenon. Health-literacy messages from healthcare providers should comprise of but not be limited to information and guidelines on infant nutrition, prevention of illnesses, hand washing and personal hygiene, exclusive breastfeeding, sanitation, immunisation and sterilisation of infants’ items.

Social-support for mothers should come during pregnancy and continue after childbirth. Information that concerns the health needs of women and their new-borns should reach the husbands since they are the main decision-makers in the home. It is however not to be limited to the husband of the woman especially for those who may not be living with their marital partners for some reason. Nursing mothers should be strengthened with skills that will make them willing, determined and confident enough to perform infant-survival skills.

Health care providers should make conscious efforts to instruct and counsel pregnant women on activities required for the survival of their coming infants in less time-consuming ways. This will equip them with knowledge on infant care before their infants arrive. In Addition, women should be empowered and occupied with activities that will improve their educational levels and economic status. Rural communities should also be equipped and facilitated to enhance infant-survival.

## Supplementary information

**Additional file 1.**

## Data Availability

The datasets used and analysed during the current study are available from the corresponding author on reasonable request.

## Abbreviations

EBF: Exclusive Breastfeeding
HIV/AIDS: Human immunodeficiency virus/acquired immune deficiency syndrome
ITNs: Insecticide Treated Nets
MDG-4: Millennium Development Goal-4
PRECEDE: Predisposing Reinforcing and Enabling Constructs in Educational Diagnosis and Evaluation
SPSS: Statistical Package for Social Sciences
