# Supplementary material for: Predictors of infant-survival practices among mothers attending paediatric clinics in Ijebu-Ode, Ogun State, Nigeria
Source: BMC Public Health. 2020 Aug 17;20:1245. doi: 10.1186/s12889-020-09310-3 (PMC7430007; doi:10.1186/s12889-020-09310-3)
Supplement: Supplementary file 1 — Additional file 1. [file 12889_2020_9310_MOESM1_ESM.docx]

**Appendix**

**Questionnaire on Predictors of infant-survival practices among mothers attending paediatric clinics in Ijebu-ode, Ogun state.**

**Dear Respondent,**

The purpose of this study is to gather essential data on **infant-survival practices** among mothers with infants attending paediatric clinics in Ijebu-Ode, Ogun State. You will be required to respond to questionnaire on what you know about infant-survival techniques. The completion of this questionnaire is voluntary. Your willingness to answer the questions implies that you have consented to participate in this study. It is required that sincere responses be given as there are no right or wrong answers. All information gathered during this study will be treated with confidentiality. Please, do not print your name.

Kindly indicate willingness to participate by ticking (√) in the BOX [ ].

Thanks for your cooperation.

**Section A: Demographic Data**

Please, provide your responses; you can only select one option for each question.

**Age** as at last birthday: ____________ years.

**Marital status**: **a.** Single [ ] **b**. Married [ ] **c**. Separated [ ] **d**. Widowed [ ] **e.** Divorced [ ]

**Occupational status:** **a.** Unemployed [ ] **b.** Self-employed [ ] **c.** Civil servant/Private organisation [ ]

**d.** Housewife [ ] **e.** Retired [ ]

**Religion:** **a**. Christianity [ ] **b**. Islam [ ] **c**. Traditional belief [ ]

**Ethnicity:** **a.** Yoruba [ ] **b.** Igbo [ ] **c.** Hausa [ ] **d.** Others; specify ________

**Educational attainment**: **a.** Non-formal [ ] **b.** Primary [ ] **c**. Secondary [ ] **d.** Tertiary [ ]

**Number of Children alive**: **a.** one child [ ] **b**. Two children [ ] **c.** More than two [ ]

**Number of infants you have ever lost:** **a.** none [ ] **b.** one [ ] **c.** two [ ] **d.** more than two [ ]

**Section B: Health-Literacy counsels received during antenatal visits**

Please, tick (√) your response as appropriate in the boxes provided to indicate what you were taught during antenatal sessions:

Infants are fragile and cannot eat just any food? **(a)** Yes [ ] **(b)** No [ ]

When the food given to an infant is handled in an unclean way, it can lead to diarrhoeal infections.

**(a)** Yes [ ] **(b)** No [ ]

*For the following, choose as many responses that apply.*

Which of the following should be prevented because it is very harmful to an infant?

(a) Bites from mosquitoes [ ]

(b) Colostrum [ ]

(c) Dirty environment [ ]

(d) Immunisation [ ]

(e) Herbal concoction (agbo) [ ]

Which of these is the best food for infants who are less than six months old?

(a) Infant formula [ ]

(b) Breast Milk [ ]

(c) Pasteurised Cow milk [ ]

For the wellness of an infant, choose the activity that is relevant from the list below.

(a) Antenatal care sessions [ ]

(b) Creating dimples [ ]

(c) Postnatal appointments [ ]

Which of these is an essential sanitary practice to ensure that the infant is protected from falling ill?

(a) Keeping the environment clean [ ]

(b) Bathing the infant with adult medicated soaps [ ]

(c) Cleaning of nipple before breastfeeding [ ]

Which of the following did you receive instructions/counsels on during antenatal care services?

(a) Use of Insecticide Treated Nets [ ]

(b) Exclusive breastfeeding [ ]

(c) Avoidance of alcohol consumption [ ]

Which of the following activities can you carry out without assistance?

(a) Administration of prescribed medication for my infant [ ]

(b) Use of insecticide-Treated Net [ ]

(b) Preparation of infant food [ ]

(d) Preparation of Oral Rehydration Solution [ ]

(e) Sterilisation of items used for your infant [ ]

**Section C: Social-Support received from significant others**

*For the following, please, tick (√) your response as appropriate in the boxes provided. You can tick only one option for each question*. Use the keys; **N=Never; R= Rarely; O=Occasionally and A=Always**

| **Statements for Consideration** | **N** | **R** | **O** | **A** |
| --- | --- | --- | --- | --- |
| I get assistance from my husband when taking my infant for immunisation. |  |  |  |  |
| When it is time to go for immunisation, my family members tell me it is not necessary for my baby. |  |  |  |  |
| How often do you have someone to assist you when taking your infant for clinic sessions? |  |  |  |  |
| I am encouraged to practice the health counsels I have received for my infant by those around me. |  |  |  |  |
| How often do you have someone else to take care of you, and your infant? |  |  |  |  |

Who is usually present with you in ensuring the welfare of your infant? (a) More than one person [ ] (b) My husband/My mother/Mother-in-law/My sibling/husband’s sibling [ ] (c) No one [ ]

**Section D: Self-efficacy to adhere to infant-survival instructions received**

Use these Keys: **SA- Strongly Agree, A- Agreed, D- Disagree** and **SD- Strongly Disagree**

| **Statements for Consideration** | **SA** | **A** | **D** | **SD** |
| --- | --- | --- | --- | --- |
| I am not confident to take my infant for clinic sessions. |  |  |  |  |
| I am willing to comply with the counsel on six months exclusive breastfeeding for my baby. |  |  |  |  |
| I am not confident enough to take my infant for immunisation. |  |  |  |  |
| From what I know about malaria, I will be careful enough to protect my infant from getting it. |  |  |  |  |
| If I have my way, I will wash my hands anytime I need to touch my infant’s items. |  |  |  |  |
| Sterilising objects before using them for my infant is inconvenient. |  |  |  |  |
| Antenatal sessions are time-consuming, I will not attend them in the future. |  |  |  |  |
| Keeping my environment clean all the time is tasking to do. |  |  |  |  |

**Section E: Self-reported Infant-Survival Practices among mothers**

Use these Keys: **N=Never; R= Rarely; O=Occasionally and A=Always**

| **Statements for Consideration** | **N** | **R** | **O** | **A** |
| --- | --- | --- | --- | --- |
| I practice six months exclusive breastfeeding for my baby(ies). |  |  |  |  |
| I make use of Insecticide Treated Nets to prevent my baby from getting malaria. |  |  |  |  |
| I take my baby for immunisation when due. |  |  |  |  |
| How often do you clean your nipples when you need to breastfeed? |  |  |  |  |
| How frequently do you sterilise the items used for your infant? |  |  |  |  |
| I take my infant for regular clinic check-ups when required. |  |  |  |  |
| I keep my environment clean to protect my baby from falling ill. |  |  |  |  |
